# Treatment of Melanoma Cells with Chloroquine and Everolimus Activates the Apoptosis Process and Alters Lipid Redistribution

**DOI:** 10.3390/ijms252212278

**Published:** 2024-11-15

**Authors:** Dorota Ciołczyk-Wierzbicka, Marta Zarzycka, Wojciech Placha, Grzegorz Zemanek, Karol Wierzbicki

**Affiliations:** 1 Chair of Medical Biochemistry, Jagiellonian University Medical College, ul. Kopernika 7, 31-034 Cracow, Poland; marta.zarzycka@uj.edu.pl (M.Z.); wojciech.placha@uj.edu.pl (W.P.); grzegorz.zemanek@uj.edu.pl (G.Z.); 2 Department of Cardiovascular Surgery and Transplantology, Institute of Cardiology, Jagiellonian University, John Paul II Hospital, ul. Prądnicka 80, 31-202 Cracow, Poland

**Keywords:** everolimus, chloroquine, apoptosis, lipids, melanoma, immunosuppressive treatment

## Abstract

The balance between apoptosis and autophagy plays a key role in cancer biology and treatment strategies. The aim of this study was to assess the effect of the mTOR kinase inhibitor everolimus and chloroquine on the regulation of proliferation, caspase-3 activation, and apoptosis in melanoma cells. We studied the activity of caspase-3 and the levels of caspase-3 and -9 using the Western blot technique. Cellular apoptosis was examined using a DNA fragmentation assay, and changes in the cell nucleus and cytoskeleton were examined using fluorescence microscopy DAPI, OA/IP. We also studied the rearrangement of lipid structures using fluorescent dyes: Nile Red and Nile Blue. A low nanomolar concentration of the mTOR kinase inhibitor everolimus in combination with chloroquine activated the apoptosis process and decreased cell proliferation. These changes were accompanied by an obvious change in cell morphology and rearrangement of lipid structures. Alterations in lipid redistribution accompanying the process of apoptosis and autophagy are among the first to occur in the cell and can be easily monitored in in vitro studies. The combination of mTOR inhibitors and chloroquine represents a promising area of research in cancer therapy. It has the potential to enhance treatment efficacy through complementary mechanisms.

## 1. Introduction

**Figure 1:**
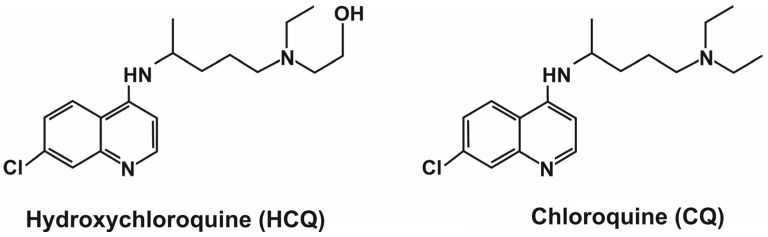
Structure of hydroxychloroquine (HCQ) and chloroquine (CQ).

Chloroquine (CQ) and hydroxychloroquine (HCQ) are 4-aminoquinoline-based (Figure 1) compounds that have been used to treat and prevent malaria caused by *Plasmodium vivax*, *P. oval*, and *P. malaria* since the 1930s [1]. These drugs also have immunomodulatory effects; hydroxychloroquine has been used for decades to treat autoimmune diseases such as systemic lupus erythematosus (SLE) and rheumatoid arthritis (RA) [2]. Chloroquine and its analog, hydroxychloroquine, have a rapid onset, long duration, low toxicity, and high tolerance in humans [1]. These drugs are currently of great interest due to their potential use in anticancer therapies, inhibition of autophagy, and sensitization of cells to the apoptosis process [3,4,5]. Chloroquine and its derivatives are promising drugs, and because of their relatively low cytotoxicity, they may allow researchers to sensitize tumors to a range of anticancer drugs [4,6].

Autophagy is an important and complex process involved in recycling cellular components. It plays a unique role in the tumor microenvironment, where the availability of nutrients is limited [7,8]. One of the most well-known effects of this drug is the inhibition of autophagy by increasing the pH of lysosomes. Recently, another mechanism of autophagy inhibition has been proposed by preventing the fusion of autophagosomes and lysosomes [6].

Serine-threonine kinase mTOR (mammalian target of rapamycin) is a major component of larger protein complexes called mTORC1, mTORC2, and mTORC3, with different cellular localizations and functions [9]. The mTORC1 complex is associated with endosomal and lysosomal membranes and initiates translation of the proteins necessary for cell cycle progression and nucleotide and lipid synthesis, and it is also an autophagy suppressor. Inhibitors of the mTORC1 complex, such as rapamycin, induce cellular autophagy [10]. In contrast, the mTORC2 complex is associated with the plasma membrane; its major functions are the organization of the cytoskeleton, cell migration, inhibition of apoptosis, and influencing cellular metabolism. More recently, the mTORC3 complex has also been mentioned, which is insensitive to rapamycin. This complex plays a role in the proliferation and drug resistance process [11].

For several decades, mTOR kinase inhibitors such as rapamycin and its derivatives, everolimus, have been used in immunosuppressive therapy after organ transplantation [12]. Then, due to their ability to regulate cellular processes, they became of interest in the context of cancer and diseases [11], as well as age-related conditions [13].

Based on the promising results of our previous studies regarding mTOR kinase inhibitors in inhibiting proliferation, the cell cycle, tumor invasion [14,15], and activation of the apoptosis process in melanoma cells [16,17], we decided to test different concentrations of everolimus in combination with chloroquine on caspase-3 activation, apoptosis, proliferation, and organization of cellular structures and lipid redistribution.

## 2. Results

### 2.1. The Effect of Chloroquine and mTOR Kinase Inhibitor Everolimus on Caspase-3 Activity and Proliferation

**Figure 2:**
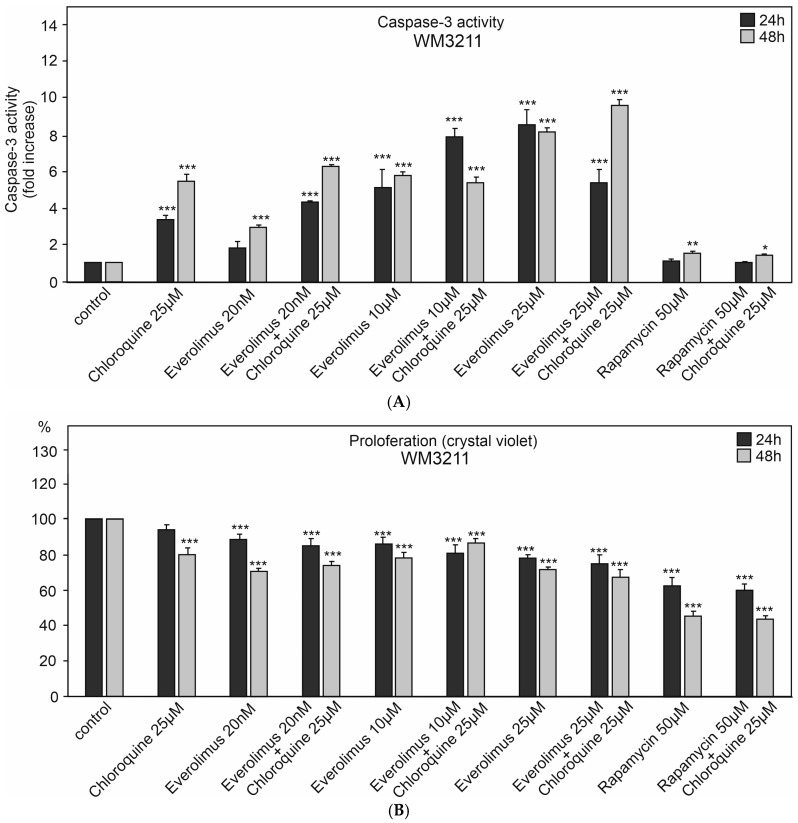
The effect of chloroquine and concentration of the mTOR kinase inhibitor everolimus on caspase-3 activity (**A**) and cell proliferation in WM3211 melanoma cell lines (**B**). Caspase-3 activity (**A**) and cell proliferation crystal violet assay (**B**) were calculated from the mean values of three independent experiments. Each value was expressed as a ratio between the caspase-3 activity or cell proliferation level and the control level. The control value was set to 1 for caspase-3 activity and 100% for cell proliferation. The data are presented as mean ± standard deviation; statistical analyses were performed using one-way ANOVA with a post hoc Dunnett test (Statistica 12.0, StatSoft, Warsow, Poland); significant differences from control, (*) *p* < 0.05, (**) *p* < 0.01, (***) *p* < 0.001.

**Figure 3:**
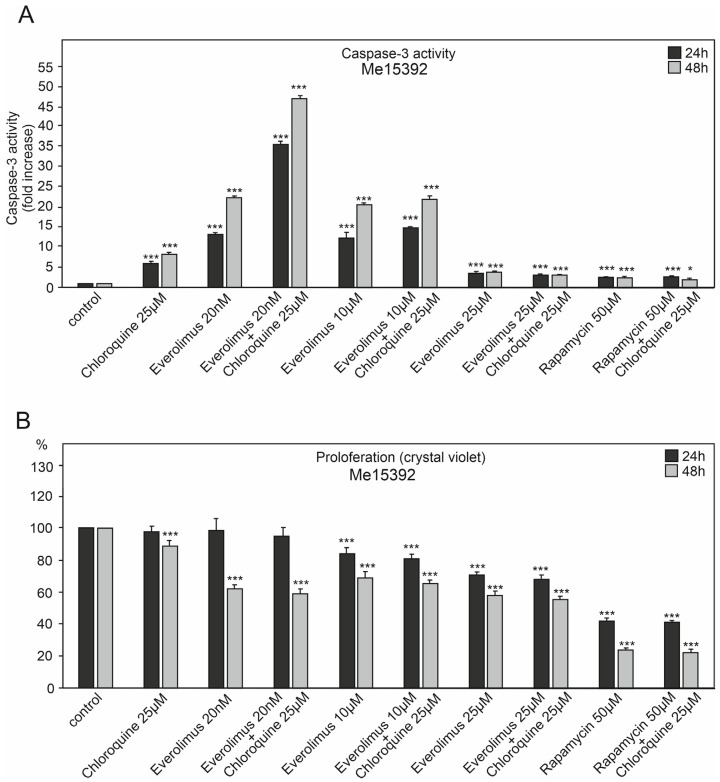
The effect of chloroquine and concentration of the mTOR kinase inhibitor everolimus on caspase-3 activity (**A**) and cell proliferation in Me15392 melanoma cell lines (**B**). Significant differences from control, (*) *p* < 0.05, (***) *p* < 0.001.

**Figure 4:**
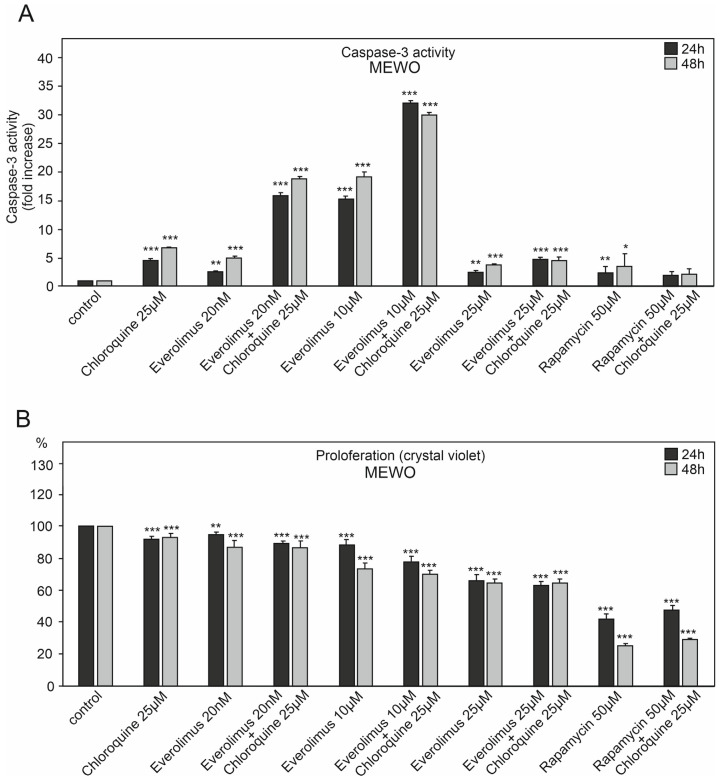

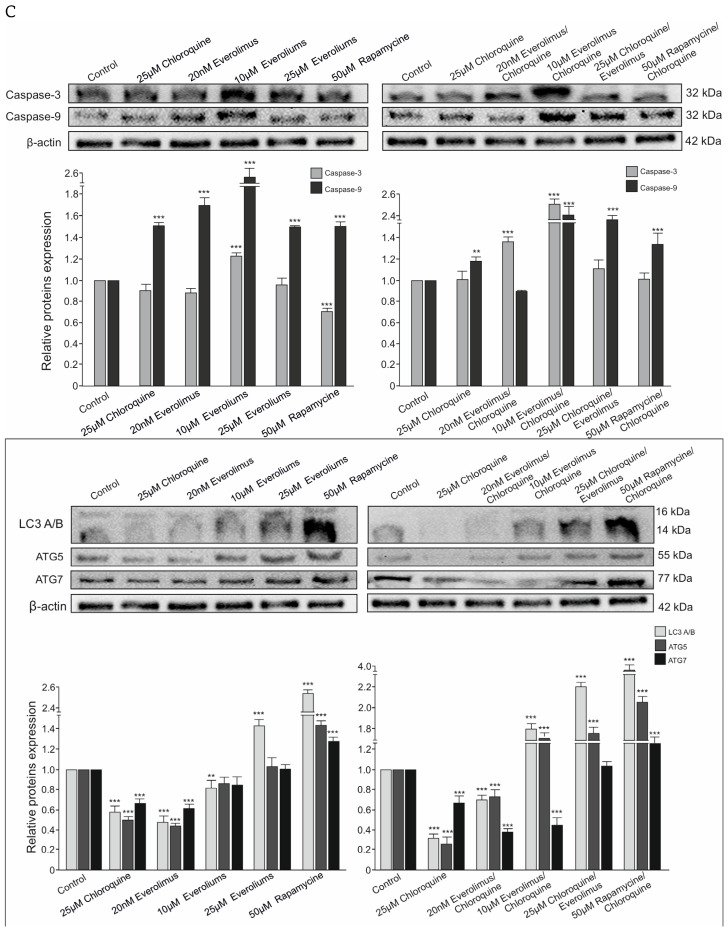
The effect of chloroquine and concentration of the mTOR kinase inhibitor everolimus on caspase-3 activity (**A**) and cell proliferation in MEWO melanoma cell lines (**B**). (**C**) The effect of chloroquine, everolimus, and rapamycin on caspase-3, caspase-9, LC3A/B, ATG-5, and ATG-7 protein levels in MEWO melanoma cell lines. Actin was used as a loading control. The densitometric analysis of protein content was normalized against its corresponding β-actin data point. The data obtained from three separate analyses are expressed as mean ± SD. Statistical analyses were per-formed using one-way ANOVA with a post hoc Dunnett test (Statistica 12.0, StatSoft); significant differences from control values are indicated as follows: (*) *p* < 0.05, (**) *p* < 0.01, (***) *p* < 0.001.

We studied the effect of chloroquine and various concentrations of everolimus (20 nM–25 μM) on caspase-3 activation and melanoma cell proliferation (Figure 2, Figure 3 and Figure 4). We also used another mTOR kinase inhibitor, rapamycin (sirolimus), at a high concentration of 50 μM, which induces autophagy (Figure 2, Figure 3 and Figure 4).

Three melanoma cell lines, primary (VGP) WM3211 and metastatic to lymph nodes Me15392 and MEWO, were used for this study.

The melanoma cell line WM3211, derived from the primary lesion (VGP) and wild type for BRAF, PTEN, N-RAS, and CDK4 and with a mutation at position 576 in the c-KIT gene, showed the lowest tendency to activate caspase-3. It was characterized by a smaller decrease in proliferation than the other melanoma cell lines (Figure 2, Figure 3 and Figure 4).

Chloroquine was characterized by a greater tendency to activate caspase-3 in melanoma cell lines for 48 h than 24 h. Caspase-3 activation for chloroquine for 24 h ranged from 3.4–6 times higher compared to the control (*p* < 0.001) (Figure 2A, Figure 3A and Figure 4A). For 48 h, these values ranged from 5.5 times for the WM3211 line, characterized by the lowest caspase-3 activations, to 8.2 times for the Me15392 line (*p* < 0.001) (Figure 2A, Figure 3A and Figure 4A).

Chloroquine for the tested melanoma cell lines showed moderate decreases in cell proliferation, ranging from 3–7% for 24 h to 20% (*p* < 0.001) for 48 h (Figure 2B, Figure 3B and Figure 4B).

Everolimus was used at three different concentrations: 20 nM, 10 μM, and 25 μM. The highest concentration of this inhibitor showed the lowest activation of caspase-3 from 2.7 to 8.4 times compared to the control (*p* < 0.001, Figure 2A, Figure 3A and Figure 4A) with a simultaneous significant decrease in cell proliferation in the range of 30–40% (*p* < 0.001, Figure 2B, Figure 3B and Figure 4B).

The melanoma cell line Me15392 showed already apparent activation of caspase-3 for the lowest 20 nM concentration, which was significantly higher for 48 h and 22 times higher in relation to the control (*p* < 0.001, Figure 3A).

A similar increase in caspase-3 activation was observed for a much higher concentration—10 μM of everolimus—in melanoma cell lines derived from metastases Me15392 and MEWO (*p* < 0.001), while for the primary melanoma cell line—WM3211—these values did not exceed 8 times that of the control (Figure 2A, Figure 3A and Figure 4A).

For low 20 nM concentrations of mTOR inhibitors, the decrease in cell proliferation was insignificant, with 10 μM higher within the range of 12% and even up to 34% (*p* < 0.001, Figure 2B, Figure 3B and Figure 4B).

For the mentioned concentrations, 20 nM and 10 μM, the combination with chloroquine clearly enhanced the effect of caspase-3 activation (Figure 2A, Figure 3A and Figure 4A), with a slight decrease in cell proliferation compared to the single agent (Figure 2B, Figure 3B and Figure 4B).

The Me15392 cell line demonstrated higher sensitivity to lower concentrations of the mTOR kinase inhibitor and chloroquine. It showed very high activation of caspase-3 over 35 times for the 24 h timeframe and as much as 47 times compared to the control for the 48 h timeframe (*p* < 0.001, Figure 3A). For the higher concentration of 10 μM everolimus in combination with chloroquine, this increase was smaller by 15–22 times for the 24 h and 48 h timeframes, respectively (*p* < 0.001, Figure 3A).

The second metastatic line, MEWO, required a higher 10 µM concentration of everolimus plus chloroquine for high caspase-3 activation, resulting in an approximately 30-fold increase over the control and less than a 20-fold increase for the 20 nM concentration of this inhibitor (*p* < 0.001, Figure 4A).

Rapamycin at the concentration of 50 μM alone or in combination with chloroquine did not cause a significant increase in caspase-3 activation (Figure 4A). However, it showed a high 60–70% decrease in cell proliferation (*p* < 0.001, Figure 4B).

The expression of caspase-3 and -9 in MEWO melanoma cell lines is presented in Figure 4C.

The highest increase in the level of caspase-3 protein by about 2.5-fold compared to the control group was obtained in the case of using a 10 μM concentration of everolimus in combination with chloroquine (*p* < 0.001, Figure 4C), and a slightly lower result was obtained for caspase-9—an increase by about 2-fold (*p* < 0.001, Figure 4C).

At the same time, high concentrations of mTOR kinase inhibitors—everolimus (25 µM) and especially 50 µM rapamycin—resulted in a significant increase in the levels of LC3 A/B, ATG-5, and ATG-7 proteins—markers of cellular autophagy. These values were 1.5- and 2.5-fold increases compared to the control for LC3 A/B proteins (*p* < 0.001, Figure 4C).

A slightly lower increase in ATG-5 and ATG-7 protein levels compared to the control, ranging from 30% to 50%, was observed for 50 µM rapamycin concentrations (*p* < 0.001, Figure 4C).

The presence of chloroquine and its combination with the lower 20 nM and 10 µM concentrations of everolimus significantly reduced the concentrations of all tested cellular autophagy markers by about 30–50%, and this effect was no longer visible for high concentrations of mTOR inhibitors, especially rapamycin (Figure 4C).

### 2.2. Effect of Chloroquine and mTOR Kinase Inhibitor Everolimus on DNA Fragmentation ELISA Assay—Detection of Apoptosis

**Figure 5:**
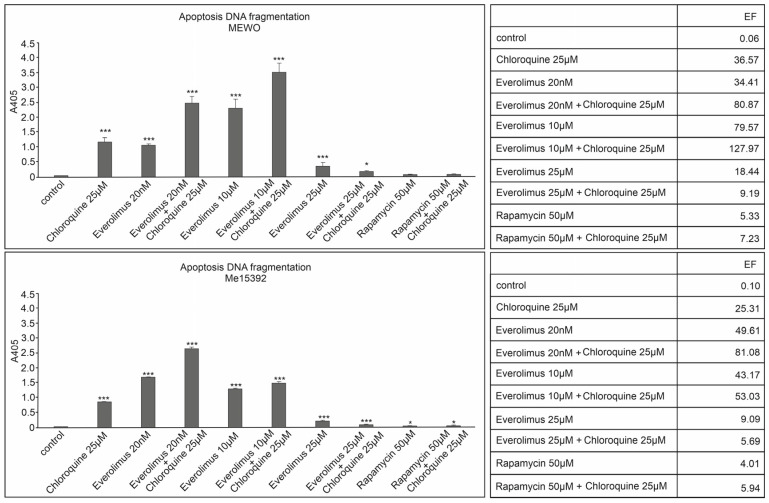
The effect of chloroquine and the mTOR kinase inhibitor everolimus on melanoma cell apoptosis MEWO and Me15392. The data are presented as mean ± standard deviation; Statistical analyses were performed using one-way ANOVA with a post hoc Dunnett test (Statistica 12.0 StatSoft); significant differences from control values are indicated as (*) *p* < 0.05, (***) *p* < 0.001. EF, enrichment factor (calculated to estimate the fold increase in DNA fragmentation in treated samples with reference to the control).

Chloroquine and the mTOR kinase inhibitor everolimus were tested in the activation of the apoptosis process in melanoma cells. The study of the induction of the apoptosis process was performed using the DNA fragmentation ELISA test for melanoma cell lines derived from metastases MEWO and Me15392, showing high caspase-3 activity. Similarly to caspase-3 activation, the melanoma cell line Me15392 showed sensitivity even at lower nanomolar concentrations of the mTOR kinase inhibitor everolimus than the MEWO melanoma cell line (Figure 5).

Both melanoma cell lines were similarly sensitive to chloroquine and showed a synergistic effect when combined with everolimus (20 nM and 10 µM). The concentration of 25 µM of everolimus did not cause a significant increase in the activation of the apoptosis process, either alone or in combination with chloroquine (Figure 5).

The highest level of apoptotic DNA degradation was observed in response to the concomitant use of chloroquine with a 20 nM concentration of everolimus for the Me15392 cell line (*p* < 0.001) and for the MEWO melanoma cell line for this combination at 10 µM concentration (*p* < 0.001) (Figure 5).

The highest level of apoptotic DNA degradation was observed in response to the simultaneous use of chloroquine with a 20 nM concentration of everolimus for the Me15392 line (*p* < 0.001) and for the melanoma cell line MEWO for this combination at a concentration of 10 µM (*p* < 0.001) (Figure 5).

The single use of the mTOR kinase inhibitor everolimus also induced a high level of apoptotic DNA degradation for the melanoma cell line Me15392, already at a concentration of 20 nM, and for MEWO at a concentration of 10 µM (Figure 5).

The apoptosis process was manifested by a significant increase in absorbance value compared to the untreated sample, with the enrichment factor (EF) (calculated to estimate the fold increase in DNA fragmentation in treated samples about control one) for the Me15392 cell line reaching 49.61 and 81.08 for the 20 nM everolimus and 20 nM everolimus combined with chloroquine (Figure 5).

Meanwhile, for the MEWO melanoma cell line, the EF values for the concentration of 10µM everolimus and its combination with chloroquine were 79.57 and 127.97, respectively (Figure 5).

### 2.3. Analysis of the Process of Cellular Apoptosis and Autophagy Using Fluorescence Microscopy

**Figure 6:**
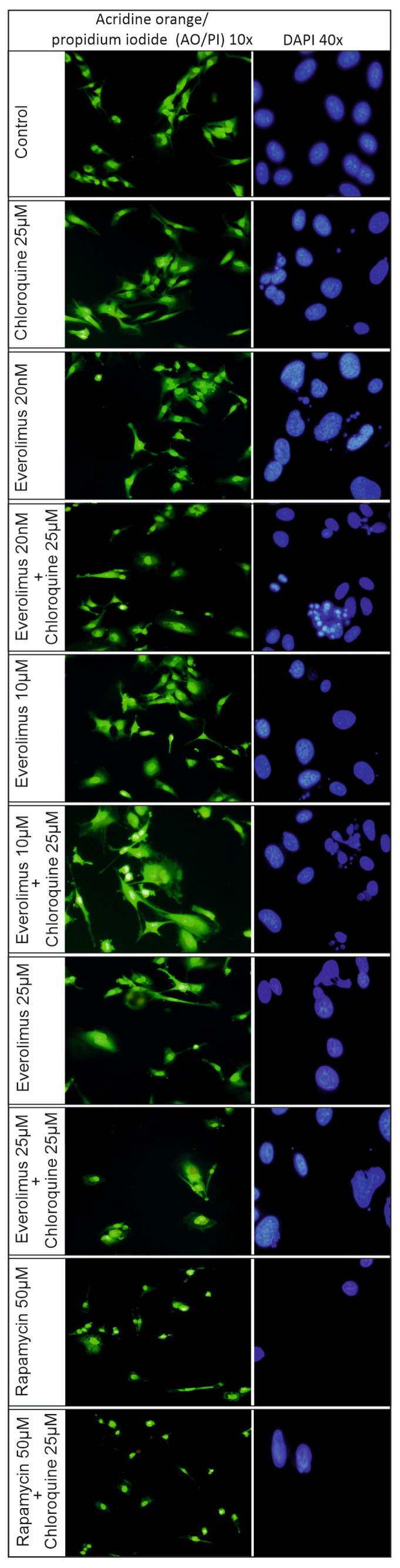
The morphological changes in Me15392 melanoma cells after treatment with chloroquine and everolimus for 24 h followed by acridine orange/propidium iodide (AO/PI) and DAPI staining. The concentrations of the inhibitors used are described in the Section 4. The experiments were performed in triplicate.

Morphological changes in melanoma cells, Me15392, after using chloroquine, 20 nM–25 μM concentrations of everolimus, and their combination with chloroquine and 50 μM rapamycin were examined using fluorescent staining: acridine orange with propidium iodide (AO/PI) and DAPI (Figure 6).

Apoptosis was confirmed by DAPI staining, which showed apparent changes in the nuclear morphology (chromatin condensation and nuclear fragmentation) of the Me15392 melanoma cells.

Similarly to the DNA fragmentation assay (Figure 5), the most visible changes in the cell nucleus (fragmentation, apoptotic bodies, chromatin condensation) were observed for 20 nM concentration of everolimus in combination with chloroquine alone (Figure 6). The 10 μM concentration of everolimus alone and in combination with chloroquine gave a less pronounced effect (Figure 6).

The changes in the cell nucleus were accompanied by changes in the morphology of the cell cytoskeleton, visible in acridine orange staining (Figure 6).

A high concentration of everolimus, 25 μM, also induced changes in the shape of the cell nucleus and a distinct effect on the shape of the cell, staining acidic cell structures in orange (Figure 6).

For a 50 μM concentration of rapamycin, no apoptotic changes were observed, only changes in the shape of the nucleus and a drastic decrease in cell proliferation (Figure 6). A noticeable change in cell shape from spindle-shaped to oval was also observed. Dead cells appeared due to propidium iodide staining (Figure 6).

### 2.4. Changes Concerning Lipid Structures

**Figure 7:**
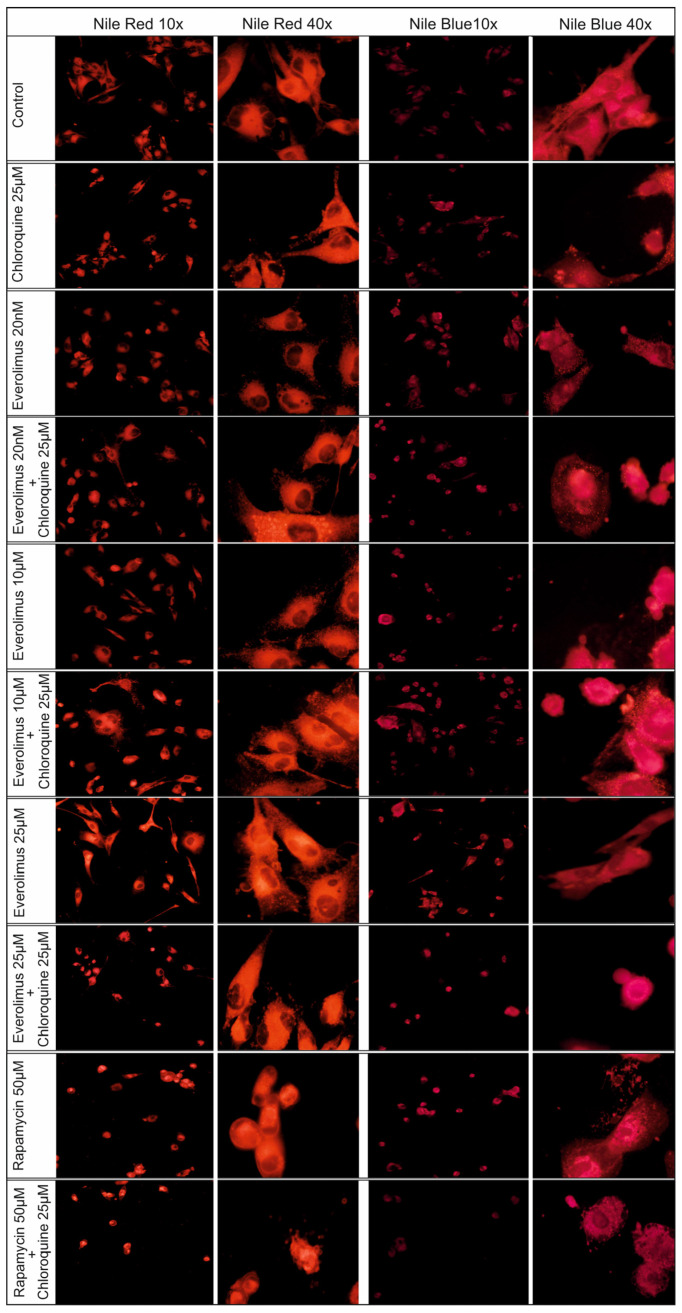
Changes concerning lipid structures of Me15392 melanoma cells after treatment with chloroquine and everolimus for 24 h followed by Nile Red and Nile Blue.

In addition to changes at the level of the cell nucleus and cytoskeleton, we also studied changes in the lipid structure. We tested Me15392 melanoma cells after treatment with chloroquine and everolimus in the concentration range of 20 nM–25 μM, alone and in combination with chloroquine and 50 μM rapamycin (Figure 7).

The following fluorescent dyes were used for the study: Nile Red and Nile Blue. Nile Red fluoresces in the presence of a broad spectrum of lipids that typically include triglycerides, cholesterol and its esters, and phospholipids [18]. Nile Blue fluoresces in the presence of unsaturated free fatty acids at least 16 carbons in length [18].

The changes in the level of lipid identification in melanoma cells compared to the control line were evident and quite similar for both staining methods used (Figure 6). Changes in the level of lipid identification in melanoma cells compared to the control line were obvious and quite similar for both staining used (Figure 7).

The presence of lipid droplets in the outline of the cell cytoskeleton creates a “lace” structure for systems in which previous tests detected cellular apoptosis (Figure 7). Using 50 µM rapamycin produced a different effect: a compact oval structure (Figure 7).

The concentrations of the inhibitors used are described in the Section 4. The experiments were performed in triplicate.

### 2.5. Cytotoxicity Assay

The cytotoxicity of dimethyl sulfoxide (DMSO) and chloroquine (25 μM), everolimus (20 nM, 10 μM, 25 μM), and rapamycin (25 μM, 50 μM) was determined using the Cytotoxicity Detection Kit LDH, Roche, Germany (Table 1 and Table 2).

Chloroquine and everolimus at the tested concentrations, individually and in combination, did not show cytotoxic effects within 24 h, 48 h, and 72 h in any of the tested melanoma cell lines. LDH activity in the medium never exceeded 2.7%, while rapamycin at a concentration of 25 μM showed an increased cytotoxicity effect within 48 h and 72 h to 5.4% and 7.1%, respectively. The 50 μM concentration of rapamycin is already cytotoxic to melanoma cells (9.1% after 24 h, 15 after 48 h, and 18% after 72 h). DMSO was used to prepare the stock solution, and even in concentrations of 1–2% it did not show any cytotoxic effect. The final concentration of DMSO did not exceed 0.05%.

## 3. Discussion

Apoptosis and autophagy are critical cellular processes that help maintain homeostasis by regulating cell death and survival. Both processes play complex and sometimes contrasting roles in cancer development and progression [19,20]. Apoptosis, or programmed cell death, is a highly regulated mechanism that allows the body to eliminate damaged, unwanted, or potentially harmful cells. It is a vital defense mechanism against cancer, as it removes cells with irreparable DNA damage, mutations, or oncogenic potential.

Due to their roles in cancer, both apoptosis and autophagy are attractive targets for cancer therapy [19]. Strategies to target these processes include therapies that restore apoptotic pathways in cancer cells.

In advanced cancers where autophagy promotes survival, autophagy inhibitors, such as chloroquine and hydroxychloroquine, are being tested in combination with other therapies to enhance the efficacy of cancer treatments.

Understanding the balance between apoptosis and autophagy in cancer cells is crucial for developing effective therapeutic strategies [21,22].

Our previous studies have shown promising results regarding the effects of mTOR kinase inhibitors on processes related to cell invasion, proliferation, cell cycle regulation, and cell invasion [14,15]. As well as activating the apoptosis process in melanoma cells [16,17], we were prompted to undertake studies using chloroquine and various concentrations of the mTOR kinase inhibitor everolimus to activate the apoptosis process in melanoma cells. We also tried to draw attention to the relationship between the process of cellular apoptosis and autophagy and the redistribution of lipids in the cell.

### 3.1. Chloroquine and Everolimus in Activating the Apoptosis Process and Inhibiting Proliferation in Melanoma Cells

Caspase-3 activation is a critical event in the execution phase of apoptosis (programmed cell death), which is central to development, immune responses, and the elimination of damaged or cancerous cells. Caspase-3 is a protease that, once activated, cleaves various substrates, leading to cell death. The activation of caspase-3 can be modulated by various cellular pathways, including those influenced by chloroquine and mTOR kinase inhibitors.

We tested three melanoma cell lines, primary (VGP) WM3211 and metastatic: Me15392 and MEWO, for 25μM concentrations of chloroquine and mTOR inhibitor everolimus in concentrations ranging from 20 nM to 25 μM and 50 μM concentrations of rapamycin alone and in combination with chloroquine.

Melanoma cell lines derived from metastases Me15392 and MEWO show high caspase-3 activity after stimulation with chloroquine and everolimus. Therefore, the activation of the apoptosis process was tested using the ELISA DNA fragmentation test for these lines. To confirm the changes in the cell cytoskeleton and cell nucleus, fluorescent staining was used: acridine orange with propidium iodide (AO/PI) and DAPI.

Studies on prostate cancer have indicated that a single administration of chloroquine at a concentration of 25 μM for cells and 50 mg/kg/day for mice induced apoptosis, playing a pivotal role in inhibiting metastatic tumor growth [3]. Our previous studies on mTOR kinase inhibitors indicated that even nanomolar concentrations of everolimus, in combination with the MEK kinase inhibitor AS-703026, strongly activated the apoptosis process in melanoma cells [16].

Chloroquine used alone resulted in an apparent activation of caspase-3 after 48 h, whereas, after 24 h, its activation was much lower; similar observations were noted by Kim et al. (2010) [23] regarding glioma cells. The observed decrease in cell proliferation after chloroquine application was not very significant, in the range of 3–7% for 24 h and a maximum of 20% for 48 h.

Chloroquine sensitized melanoma cells to the action of everolimus, enhancing caspase-3 activation in a synergistic manner compared to a single application of this inhibitor. Similar results were obtained regarding the combined treatment of osteosarcoma cells with rapamycin and chloroquine [24]; chloroquine enhances apoptotic cell death by blocking autophagy.

Similarly to caspase-3 activity, the Me15392 line showed greater sensitivity to lower concentrations of the mTOR kinase inhibitor everolimus already at nanomolar levels, while for the MEWO line, this concentration had to be as low as 10 μM. The combination of chloroquine with everolimus in nanomolar concentrations for the Me15392 melanoma cell line and 10 μM for the MEWO cell line synergistically increased the apoptosis process’s activation. The apoptosis process was confirmed by the ELISA DNA fragmentation test and in the microscopic image concerning changes in the cell nucleus and cytoskeleton.

Fluorescent staining of the cell nucleus with DAPI showed visible chromatin condensation and the formation of apoptotic bodies. Changes in the cell cytoskeleton were also confirmed by staining with acridine orange and propidium iodide. Characteristic formation of vesicular protrusions through the plasma membrane has been observed as a result of cytoskeleton shrinkage [25,26].

Similar observations were also made by Grimaldi et al. 2015 [27] regarding the use of chloroquine and everolimus in renal cell carcinoma therapy. Renal cancer cells showed different sensitivity to chloroquine and everolimus. In contrast, the combination of both drugs showed a strong synergistic effect of inhibiting cell proliferation and activating the apoptosis process while inhibiting autophagy for relatively high micromolar concentrations of everolimus. Grimaldi et al. (2015) [27] also confirmed that the apoptosis effect induced by the combination of micromolar concentrations of chloroquine and everolimus resulted in the intrinsic activation of the mitochondrial apoptosis pathway. In contrast, the extrinsic pathway was only partially engaged after its activation by chloroquine. A phase I/II clinical trial based on three centers using hydroxychloroquine 600 mg and everolimus 10 mg daily in patients with advanced clear-cell renal cell carcinoma (cc RCC) showed that hydroxychloroquine at this dose inhibits autophagy and can be safely combined with everolimus [28].

A high concentration of the mTOR kinase inhibitor rapamycin at 50 μM did not induce caspase activity and apoptosis but significantly reduced (60–70%) cell proliferations. Cells had a clearly changed shape, which suggested activation of processes related to cytotoxicity and cellular autophagy. In the microscopic image, single cells stained with propidium iodide were visible. The process of cellular autophagy was also confirmed by a significant increase in the expression of autophagy markers: LC3 A/B, ATG-5, and ATG-7.

Similar observations were also made by researchers in human neuro-blastomas cells or fibroblasts [29], and many studies indicate that high 50–100 μM concentration of mTOR kinase inhibitors, especially rapamycin, induces autophagy [30].

The research group Juridic et al. (2022) noticed an interesting observation regarding long-term overprotection due to increased intestinal autophagy during short-term treatment with rapamycin in an animal model [31].

The 25 μM concentration of everolimus activated the apoptosis process in a small percentage of cases. According to [24], the use of 20 μM chloroquine together with a 20 μM concentration of rapamycin enhanced the apoptotic effect in MG63 osteosarcoma cells, while rapamycin promotes autophagy.

First-generation mTOR kinase inhibitors, everolimus, temsirolimus, and rapamycin, have found wide application in clinical cancer therapies, but drug resistance limits their efficacy [32]. One of the proposed mechanisms responsible for drug resistance is the increase in autophagy by inhibiting the mTORC1 complex, especially when using high concentrations of these inhibitors. Cellular autophagy is mainly considered a cytoprotective survival mechanism, in which cytoplasmic components are recycled to generate metabolic intermediates and energy.

Everolimus and temsirolimus mainly cause cytoprotective effects, while rapamycin enhances the impact of cytotoxicity [32]. Based on studies conducted by many centers [32,33], the mechanism of the autophagy and apoptosis process depends on both the drug used and the sensitivity of the given cell line.

Autophagy inhibitors are currently being studied to reduce drug resistance [34]. Clinical improvement in the efficacy of autophagy inhibition has been demonstrated by using chloroquine or hydroxychloroquine with mTOR kinase inhibitors.

### 3.2. Redistribution of Lipid Structures in Fluorescence Microscopy Images After Chloroquine and Everolimus Treatment

Lipid metabolism is often disturbed in cancer cells. These molecules are crucial for cell membrane formation, signaling, and energy storage [35]. They play a significant role in the development and progression of cancer and resistance to treatment [36,37].

One of the first changes observed during the activation of apoptosis and autophagy is the change in lipid redistribution [38].

In this work, in addition to the synergistic effect of chloroquine with low concentrations of the mTOR kinase inhibitor everolimus in activating the apoptosis process and inhibiting cell proliferation, we also wanted to show the dynamics of these changes at the level of lipid redistribution in the cancer cell.

Chloroquine is an antimalarial drug that increases the pH in acidic compartments such as lysosomes [39]. This leads to lysosomal dysfunction, which has downstream effects on autophagy, lipid metabolism, and lipid storage. Chloroquine inhibits the fusion of autophagosomes and lysosomes, leading to the accumulation of autophagosomes [40,41]. Lipids, especially in the form of lipid droplets, can be sequestered in these autophagy vesicles.

Chloroquine can also impair cholesterol transport and promote the accumulation of lipid species, particularly phospholipids, in lysosomes.

We observed significant changes in fluorescence microscopy images of melanoma cells stained with Nile Red and Nile Blue and treated with 25 μM chloroquine.

The fluorescent dye Nile Red has been used to stain intracellular lipid droplets in a hydrophobic environment. This dye exhibits intense fluorescence in all organic solvents, staining intracellular lipids and lysosomal phospholipid inclusions. Its colors range from golden yellow to red [18].

The second dye, Nile Blue, shows a stringent specificity for unsaturated fatty acids with more than 16 carbon atoms in the molecule [18], and it is also sensitive to pH changes around physiological pH, showing a color from blue to dark pink [42].

Everolimus, an mTOR kinase inhibitor already at low nanomolar concentrations, induced distinct changes in the image of lipid structures. These changes were intensified after the use of a combination with chloroquine and mTOR kinase inhibitors, such as everolimus and rapamycin. These drugs inhibit the mTOR-related signaling pathway and play a pivotal role as central regulators of cell growth, proliferation, metabolism, and survival, as well as lipid and protein metabolism.

The mTORC1 complex promotes lipid synthesis by activating transcription factors and enzymes involved in lipid biosynthesis [43]. It drives de novo lipid synthesis by increasing the activity of sterol regulatory element binding proteins (SREBPs), a family of transcription factors regulating the genes synthesizing fatty acids, cholesterol, and phospholipids [44]. This complex also controls the storage and utilization of lipids, regulating processes such as fatty acid oxidation and lipid droplet formation.

The mTORC2 complex, on the other hand, is involved in lipid metabolism and affects lipid storage, mainly by regulating AKT signaling. AKT stimulates lipid uptake and fatty acid storage [44].

Alterations in lipid redistribution in cells associated with the process of cellular apoptosis and autophagy, stained with Nile Red and Nile Blue dyes, may serve as an additional rapid prognostic marker.

## 4. Materials and Methods

### 4.1. Cell Culture

The presented study used three melanoma cell lines: primary (VGP)—WM3211 and metastatic—MEWO and Me15392. WM3211 human primary melanoma cell line (VGP) has metastasis competence, wild type for *BRAF*, *PTEN*, *N-RAS*, and *CDK4*, and with a mutation at position 576 in the *c-KIT* gene. MEWO, human malignant melanoma cell line is derived from the lymph nodes. This cell line is the wild type for *BRAF*, *PTEN*, and *N-RAS*. Me15392, human malignant melanoma cell line, is derived from the lymph nodes (V600E mutation in the *BRAF* gene).

The cells were cultured in RPMI-1640 medium supplemented with 10% fetal bovine serum and antibiotics (penicillin and streptomycin). The cells were incubated at 37 °C in a humidified atmosphere of 5% CO_2_ in air. The cells were treated with chloroquine (Sigma, Darmstadt, Germany) at 25 μM concentration, everolimus (Selleck, Houston, TX, USA) at 20 nM, 10 μM, and 25 μM concentration, and rapamycin (Selleck, USA) at 50 μM concentration. The incubation times of melanoma cells with inhibitors was 24 and 48 h, respectively. The melanoma cell lines were obtained from the ESTDAB Melanoma Cell Bank (Tübingen, Germany).

### 4.2. Caspase-3 Activation Assay

The activation of caspase-3 in response to the applied inhibitors was estimated using the fluorogenic substrate DEVD-AFC (Biovision, Milpitas, CA, USA), described previously [16].

### 4.3. Western Blot Analysis

The sample was prepared for SDS-PAGE electrophoresis as described previously [45]. Antibodies against A2156 caspase-3 Rabbit pAb (ABclonal, Düsseldorf, Germany), A18676 caspase-9 Rabbit mAb (ABclonal, Germany), 4108 LC3 A/B (Cell Signaling Technology, Danvers, MA, USA), A19677 ATG-5 Rabbit pAb (ABclonal, Germany), A19604 ATG-5 Rabbit pAb (ABclonal, Germany), and β-actin (A2228, SIGMA, Düsseldorf, Germany) were used to detect indicated proteins. Bands were visualized using horseradish peroxidase—coupled with secondary anti-mouse or anti-rabbit antibodies (Cell Signaling Technology, Danvers, MA, USA). Protein immunoreactivity was detected using a chemiluminescence method, and images were recorded using the ChemiDoc MP imaging system (Bio-Rad Labs, Hercules, CA, USA). To obtain quantitative results, immunoblots were scanned using SynGene Gene Tools version 4.03.0 (Synoptics Ltd. Beacon House, Nuffield Road, Cambridge, CB4 1TF, UK). Densitometry was used to normalize to β-actin protein level. Representative membranes from at least three independent experiments with similar results are presented in the figures.

### 4.4. DNA Fragmentation ELISA Assay

The induction of apoptosis was confirmed using an enzyme-linked immunosorbent assay described previously [16], assessing the intracellular level of cytoplasmic histone-associated DNA fragments (mono- and oligonucleotides) that appear after activation of caspase-dependent endonucleases.

### 4.5. Cell Proliferation

Cell proliferation was assessed with the crystal violet test, as previously described [16].

### 4.6. Cytotoxicity Assay

Cytotoxicity of dimethyl sulfoxide (1–2%) (DMSO), chloroquine (1–25 μM), and mTOR kinase inhibitors—everolimus (20 nM–25 μM) and rapamycin (sirolimus) 25–50 μM—was determined using Cytotoxicity Detection Kit LDH, Roche, Berlin, Germany. Cytotoxicity was tested in the concentration ranges used individually and in combination with chloroquine. DMSO was used to prepare the stock solution, and the maximum DMSO concentration did not exceed 0.05%.

### 4.7. Fluorescence Microscopy

Melanoma cells were visualized using a fluorescent microscope from the Olympus Corporation, Tokyo, Japan.

### 4.8. DAPI Staining Assay

4′,6-Diamidine-2′-phenylindole dihydrochloride (DAPI, Roche, Berlin, Germany) staining was carried out according to the method described by manual protocol Cat. No. 10 236 276 001 (Roche, Germany). Melanoma cells were visualized using a fluorescent microscope from the Olympus Corporation, Tokyo, Japan.

### 4.9. Acridine Orange (AO)/Propidium Iodide (PI) Live and Dead Cell Co-Staining

Acridine orange (AO)/propidium iodide (PI) double staining was carried out according to the method described in the manual protocol Kit K2238 (APEx BIO, Boston, MA, USA).

Melanoma cells were visualized using a fluorescent microscope from the Olympus Corporation, Tokyo, Japan.

### 4.10. Nile Red Staining Assay

Nile Red cell staining was performed based on the method [18,46] described.

Melanoma cells were stained with a 10 μM solution of Nile Red (Carl Roth, Karlsruhe, Germany) in a culture medium for 10 min at 37 °C in a humidified atmosphere of 5% CO_2_. The solution was then gently removed, and the cells were washed twice in a culture medium. The fluorescence of cellular lipids was measured at the excitation and emission wavelengths of 559 nm/635 nm, respectively, using a fluorescence microscope (Olympus Corporation, Tokyo, Japan).

### 4.11. Nile Blue Staining Assay

Nile Blue cell staining was performed based on the method [18,46,47] described.

Melanoma cells were stained with a 5 μM solution of Nile Blue (Carl Roth, Karlsruhe, Germany) in a culture medium for 10 min at 37 °C in a humidified atmosphere of 5% CO_2_. The solution was then gently removed, and the cells were washed twice in a culture medium. The fluorescence of cellular lipids was measured at the excitation and emission wavelengths of 628 nm/660 nm, respectively, using a fluorescence microscope (Olympus Corporation, Tokyo, Japan).

### 4.12. Statistics

The data on caspase-3 activity, proliferation, and apoptosis were calculated from the mean values of repeated experiments. The statistical analysis was conducted using one-way ANOVA coupled with a post hoc Dunnett test using Statistica 12.0, StatSoft, Poland. The statistical significance is presented in the pertinent figures.

## 5. Conclusions

In summary, both apoptosis and autophagy play critical roles in cancer treatment strategies, and ongoing research aims to understand their roles better and find optimal ways to use them in anticancer therapy.

Understanding the specific roles of apoptosis and autophagy in a particular type of cancer may help increase therapy’s efficacy and personalization.

Therapies based on mTOR kinase inhibitors and chloroquine seem promising. They induce the apoptosis process and modulate cellular autophagy, which may provide a synergistic effect, improving the overall response to treatment.

## Figures and Tables

**Table 1:** Cytotoxicity of DMSO in melanoma cell lines.

		Control	DMSO 1%	DMSO 2%
Wm3211	24 h	1.1 ± 0.1	1.0 ± 0.1	1.1 ± 0.1
	48 h	1.5 ± 0.1	1.4 ± 0.1	1.6 ± 0.1
	72 h	1.8 ± 0.1	1.8 ± 0.2	2.0 ± 0.1
Me15392	24 h	2.1 ± 0.1	2.2 ± 0.1	2.1 ± 0.1
	48 h	2.3 ± 0.1	2.4 ± 0.1	2.1 ± 0.1
	72 h	2.4 ± 0.1	2.5 ± 0.1	2.6 ± 0.2
MEWO	24 h	1.8 ± 0.1	2.0 ± 0.2	2.1 ± 0.1
	48 h	2.0 ± 0.1	2.2 ± 0.2	2.1 ± 0.2
	72 h	2.1 ± 0.1	2.2 ± 0.2	2.3 ± 0.1

**Table 2:** Cytotoxicity of everolimus, rapamycin, and chloroquine in melanoma cell lines.

		Control		Everolimus 20 nM		Everolimus 10 μM		Everolimus 25 μM		Rapamycin 25 μM		Rapamycin 50 μM	
	25 μM Chloroquine	−	+	−	+	−	+	−	+	−	+	−	+
	Cytotoxicity [%]												
Wm3211	24 h	1.1 ± 0.1	1.2 ± 0.1	1.5 ± 0.1	1.5 ± 0.2	2.1 ± 0.1	2.0 ± 0.1	2.3 ± 0.1	2.3 ± 0.1	3.2 ± 0.2	3.0 ± 0.2	6.2 ± 0.2	5.9 ± 0.2
	48 h	1.5 ± 0.1	1.4 ± 0.1	1.7 ± 0.2	1.7 ± 0.1	2.3 ± 0.1	2.1 ± 0.1	2.3 ± 0.2	2.3 ± 0.2	4.4 ± 0.2	4.0 ± 0.2	7.2 ± 0.2	7.0 ± 0.2
	72 h	1.8 ± 0.1	1.7 ± 0.1	1.9 ± 0.2	2.0 ± 0.2	2.4 ± 0.1	2.4 ± 0.2	2.6 ± 0.2	2.4 ± 0.2	5.7 ± 0.2	5.2 ± 0.2	9.8 ± 0.2	9.1 ± 0.3
	24 h	2.1 ± 0.1	2.2 ± 0.2	2.1 ± 0.1	2.4 ± 0.1	2.4 ± 0.1	2.1 ± 0.2	2.3 ± 0.1	2.1 ± 0.1	4.2 ± 0.2	3.5 ± 0.2	8.7 ± 0.2	8.1 ± 0.2
	48 h	2.3 ± 0.1	2.1 ± 0.1	2.3 ± 0.1	2.1 ± 0.1	2.1 ± 0.1	2.0 ± 0.2	2.3 ± 0.1	2.2 ± 0.1	5.1 ± 0.2	4.5 ± 0.2	12 ± 0.3	11 ± 0.3
	72 h	2.4 ± 0.1	2.3 ± 0.2	2.2 ± 0.2	2.2 ± 0.2	2.3 ± 0.1	2.4 ± 0.2	2.6 ± 0.1	2.2 ± 0.1	6.9 ± 0.2	6.2 ± 0.1	15 ± 0.3	14 ± 0.3
MEWO	24 h	1.8 ± 0.1	1.5 ± 0.1	2.1 ± 0.2	2.4 ± 0.1	2.2 ± 0.1	2.3 ± 0.2	2.4 ± 0.1	2.3 ± 0.2	4.8 ± 0.2	3.9 ± 0.2	9.1 ± 0.3	8 ± 0.2
	48 h	2.0 ± 0.1	1.8 ± 0.1	2.0 ± 0.2	2.4 ± 0.2	2.1 ± 0.1	2.1 ± 0.2	2.1 ± 0.2	2.2 ± 0.1	5.4 ± 0.1	4.8 ± 0.2	15 ± 0.3	13 ± 0.3
	72 h	2.1 ± 0.1	2.2 ± 0.2	2.1 ± 0.2	2.3 ± 0.2	2.2 ± 0.1	2.2 ± 0.2	2.7 ± 0.2	2.1 ± 0.1	7.1 ± 0.2	6.0 ± 0.2	18 ± 0.3	15 ± 0.3

## Data Availability

The data presented in this study are available on request from the corresponding author.
